# Comparison and analysis of the efficacy and safety of minimally invasive surgery and craniotomy in the treatment of hypertensive intracerebral hemorrhage

**DOI:** 10.12669/pjms.343.14625

**Published:** 2018

**Authors:** Jixin Zhang, Shiyong Lu, Suzhen Wang, Naiyun Zhou, Guoliang Li

**Affiliations:** 1 Jixin Zhang, MD. Department of Neurosurgery, Shandong Jiyang Public Hospital Ji’nan, Shandong, China 251400; 2 Shiyong Lu, MD. Department of Neurosurgery, Shandong Jiyang Public Hospital Ji’nan, Shandong, China 251400; 3 Suzhen Wang, MD. Department of Neurosurgery, Shandong Jiyang Public Hospital Ji’nan, Shandong, China 251400; 4 Naiyun Zhou, MD. Department of Women’s Health Care, Jiyang Women and Children Baojian Railway Station Ji’nan, Shandong, China 251400; 5 Guoliang Li, MD. Department of Neurosurgery, Qingdao Chengyang District People’s Hospital, Qingdao, China 266109

**Keywords:** Cerebral hemorrhage, Minimally invasive surgery, Craniotomy

## Abstract

**Objective:**

This study was aimed to compare and analyze the effects and safety of minimally invasive and craniotomy in the treatment of hypertensive intracerebral hemorrhage.

**Methods:**

A total of 130 patients with hypertensive intracerebral hemorrhage were recruited. The patients were randomly divided into two groups (research and control group). Research group was treated with endoscopic minimally invasive surgery, while control group was treated with craniotomy and hematoma clearance. The basic situation, clinical effects, prognosis, nerve function and inflammatory factors of the two groups were compared while the condition of postoperative complications was also observed.

**Results:**

The operative time of patients in research group showed statistically significant (P<0.05) difference when compared with control group. Hematoma clearance rate and intraoperative blood loss of research group was significantly better than control group. There was no significant difference (P>0.05) between the two groups in preoperative hemorrhage and edema around the hematoma, however hemorrhage and edema around the hematoma after four weeks of surgery in the research group was significantly (P<0.05) lower than control group. After four weeks of treatment, the BI and SSS score, SP and IL-2 level of the research group were significantly higher than control group (P<0.05), while MRS score, IL-6, hs-CRP, TNF-α and SF was significantly lower than control group (P<0.05).

**Conclusion:**

Compared with craniotomy, minimally invasive surgery is more effective in the treatment of hypertensive intracerebral hemorrhage, as well as it is more conducive to restore neurological function, improve prognosis and reduce serum inflammatory factor levels.

## INTRODUCTION

Hypertensive cerebral hemorrhage is a spontaneous intracerebral hemorrhage, which is caused by hypertension and the main pathological basis is hypertension and arteriosclerosis.^1^

Arteriosclerosis results in thickening of artery intima and atheromatous plaque, which makes the lumen relatively narrow. Besides, the fibrosis of the elastic layer of the middle layer of the arteriole and the glassy change will increase the fragility of the tube wall. When the blood pressure fluctuates violently, the damaged vessels cannot be automatically adjusted, and it can be easily broken by high blood pressure, or form tiny aneurysms at the weakest part, which will cause bleeding for a long time.^2^,^3^ There are about 2.5 million patients with cerebral hemorrhage each year in the world, with 30% of the patients have cerebral hemorrhage caused by hypertension and the rate of disability is as high as 40%. The common treatment of hypertensive cerebral hemorrhage is surgery, and the treatment technology of the disease has also improved greatly through the accumulation of extensive clinical trial and treatment experience. However, there are a variety of surgical procedures, and the prognosis is not the same.^4^,^5^ The traditional surgical treatment of hypertensive cerebral hemorrhage is craniotomy which has good effect on removing hematoma and hemostasis. However, craniotomy often damages the surgical approach and the surrounding normal brain tissues, subsequently reduce the efficacy of prognosis of the patients.^6^ With the advancement of minimally invasive endoscopic technique, endoscopic surgery has good application prospectives.^7^ Our objective was to compare and analyze the effects and safety of minimally invasive and craniotomy in the treatment of hypertensive intracerebral hemorrhage.

## METHODS

A total of 130 patients with hypertensive intracerebral hemorrhage treated in our hospital from January 2016 to April 2017 were enrolled as research subjects, including 70 males and 60 cases of females, aged 56 to 68 years with a mean age of 62.4±2.8 years and intracranial hematoma of39.5±4.2 ml.

### The bleeding site

80 cases of lobar hemorrhage, 50 cases of basal ganglia hemorrhage; Glasgow coma score: 78 cases <5 points, 5 ~8 points in 42 cases, 9 ~13 points in 10 cases.

The patients were divided into two groups as research and control group according to the random number table method, with 65 cases in each group (n=65).

### Ethical Approval

The study was carried out as per Helsinki declaration and it was approved from the institutional ethical board of Shandong Jiyang Public Hospital Ji’nan, Shandong, China. The Reference number is “JAN152017/P”.

### Treatment protocol

The control group was treated with traditional craniotomy. The surgeons used craniotomy with small bone window for microscopic examination and did not damage the blood vessels and brain functional areas. In the hematoma near the scalp to make a cut from the mouth, the incision was horseshoe-shaped and the dura was radioactive. Then cut the cortex into the hematoma cavity, removed the hematoma under the microscope and sutured the dura after the removal. The bone flap decompression was performed in patients with swelling of the brain after the removal of hematoma. The drainage tube was routinely placed in the cavity of hematoma and the drainage tube was removed 3~5 days after the operation.^8^

The patients in the research group were treated with neuroendoscopic minimally invasive surgery on the basis of conventional treatment. CT examination was performed before the operation, and the CT plane with the most hematoma was selected as the cranial drilling position and the hematoma center was nearest to the intracranial plate. Using a milling cutter to mill the bone flap and cut off the dural cross, the electrocoagulation of the local cortical brain tissue was made using the bipolar electrocoagulation, and it was carefully cut at the same time.

### Observational index

The basic conditions of surgery in the two groups with hemorrhage before and after operation and edema around the hematoma were recorded, including the operation time, the hematoma clearance rate and the amount of bleeding during the operation. Three months after treatment, the prognosis of the patients was determined by the Glasgow prognosis scale: Grade-I is death; Grade-II is incontinence and bed coma; Grade-III is severe disability, and personal life needs to be taken care of by others; Grade-IV for patients to be able to take care of their own ability; Grade V for patients with good recovery, life, work and learning self-care.

Four weeks after treatment, patients were scored by Navier Stroke Scale and Barthel Index. The higher the score, better the recovery of neurological function. In contrast, the patients were scored by the Rankin Scale and the higher the score, the worse the recovery of neurological function.

5mL of venous blood before and after treatment was drawn, then the levels IL-2, IL-6, TNF-α and hsCRP were detected. SP and SF were measured before and after treatment in both groups. Subsequently, the postoperative complications of the two groups were observed and recorded.

### Statistical analysis

SPSS (v=18) was used for statistical description. Measurement data was expressed as mean ± standard deviation (X̄±s) and was analysed using student t test. The counting data was expressed by frequency and percentage and were conducted using the Chi-square test. Rank data was detected by rank sum test (U). P<0.05 means a significant difference.

## RESULTS

### Comparison of the basic and clinical effects

The operative time of patients in research group showed statistically significant difference when compared with control group (P<0.05). Hematoma clearance rate and intraoperative blood loss of the patients in the research group was significantly (P<0.05) better than control group. There was no significant difference between the two groups in preoperative hemorrhage and edema around the hematoma, but the situation of hemorrhage and edema around the hematoma within four weeks after surgery in the research group was significantly lower than control group (P<0.05) as shown in Table-I.

**Table-I:** Comparison of the basic and clinical effects between the two groups (X̄±s).

Groups	*N*	Basic condition of operation			Bleeding condition(mL)				Edema around the hematoma(mL)			

		Operative time (h)	Hematoma clearance rate (%)	Blood loss during operation(V/mL)	Preoperative	4 weeks after surgery	t value	P	Preoperative	4 weeks after surgery	t value	P
Research group	65	1.86±0.65	83.43±4.67	61.06±8.65	39.07±6.17	8.62±1.1	10.87	<0.05	16.15±1.68	5.74±1.36	12.076	<0.05
Control group	65	5.54±1.03	72.78±9.35	76.25±10.12	39.03±6.14	17.41±3	11.15	<0.05	16.19±1.52	10.13±2.3	9.583	<0.05
*t* value		7.337	8.751	9.673	0.542	7.006			0.147	9.132		
*P*		<0.05	<0.05	<0.05	0.644	<0.05			0.781	<0.05		

### Comparison of prognosis efficacy

Table-II shows no Grade I in two groups, however, the number of Grade II and III in research group was significantly lower (P<0.05) than control group, while the number of Grade IV and V in study group was significantly higher (P<0.05) than control group.

**Table-II:** Comparison of prognosis efficacy between the two groups [cases (%)].

Groups	*n*	Grade I	Grade II	Grade III	Grade IV	Grade V
Research group	65	0(0)	2(3.1)	4(6.2)	32(49.2)	27(41.5)
Control group	65	0(0)	7(10.8)	19(29.2)	24(36.9)	15(23.1)
U value	2.956					
P	<0.05					

### Comparison of neurological function scores

There was no significant difference (P>0.05) in BI, SSS and MRS score before treatment in the two groups. Detailed scores of both groups have been shown in Table-III.

**Table-III:** Comparison of neurological function scores between the two groups (X̄±s).

Groups	*n*	BI score		SSS score		MRS score	

		Prior treatment	4 weeks after treatment	Prior treatment	4 weeks after treatment	Prior treatment	4 weeks after treatment
Research group	65	19.14±6.62	68.85±11.06	10.32±2.03	36.63±7.34	20.53±3.23	6.32±1.22
Control group	65	19.21±6.13	51.62±11.12	10.38±2.21	27.52±5.95	20.16±4.06	9.46±1.35
*t* value	0.175	5.025	0.239	4.168	0.336	8.542	
*P*	>0.05	<0.05	>0.05	<0.05	>0.05	<0.05	

### Comparison of inflammatory factors

There was no significant difference in IL-2, IL-6, hs-CRP and TNF-α in the two groups before treatment (P>0.05), however they were all effectively improved four weeks after treatment. The detailed levels of inflammatory factors are shown in Table-IV.

**Table-IV:** Comparison of levels of inflammatory factors in the serum of two groups (X̄±s).

Groups	*n*	IL-2(pg/L)		IL-6(ng/L)		hs-CRP(mg/L)		TNF-a(ag/L)	

		Prior treatment	4 weeks after treatment	Prior treatment	4 weeks after treatment	Prior treatment	4 weeks after treatment	Prior treatment	4 weeks after treatment
Research group	65	34.14±5.03	46.15±5.1	45.77±4.6	13.11±2.26	16.16±3.1	6.09±2.36	63.06±4.08	39.66±3.17
Control group	65	34.55±5.3	40.21±4.3	44.24±4.5	27.27±3.04	16.13±3.1	11.43±2.0	63.02±4.12	53.37±3.32
*t* value		0.315	6.094	0.715	9.571	0.138	7.464	0.276	11.640
*P*		>0.05	<0.05	>0.05	<0.05	>0.05	<0.05	>0.05	<0.05

### Comparison of serum ferritin (SF) and serum P substance (SP

There was no significant difference (P>0.05) in SF and SP between the two groups before treatment. SF and SP both were effectively improved in the four weeks after treatment, however SF in the research group was significantly lower (P<0.05) than the control group, while SP was significantly higher (P<0.05) than that in the control group as shown in Table-V.

**Table-V:** Comparison of SF and SP between the two groups (X̄±s).

Groups	n	SF(ng/mL)				SP(pg/mL)			
		Prior treatment	4 weeks after treatment	*t* value	*P*	Prior treatment	4 weeks after treatment	*t* value	*P*
Research group	65	428.32±18.7	308.12±21.6	11.136	<0.05	14.96±2.02	28.13±3.15	8.146	<0.05
Control group	65	429.15±18.5	392.16±22.4	7.752	<0.05	15.01±1.68	18.62±2.37	8.533	<0.05
t value	0.187	12.064			0.602	9.573			
P	>0.05	<0.05			>0.05	<0.05			

## DISCUSSION

It is necessary to decompress the bone flap, electrocoagulate the cerebral cortex and separate the stretch of the brain tissue. Moreover, the brain tissue was severely damaged during the operation, and the brain edema was aggravated and the infection rate increased after operation. The results of the comparative study in this group show that operation time, hematoma clearance rate, and the amount of intraoperative bleeding, postoperative bleeding, postoperative hematoma around edema, prognosis, neurological function score and postoperative complications of patients in the research group were significantly better than the control group, and the difference between groups was statistically significant. The results of this study are similar to those in related literatures.^9^ The study suggests that neuroendoscopic minimally invasive surgery is superior to craniotomy and evacuation of hematoma for hypertensive intracerebral hemorrhage in terms of efficacy and safety.

Studies have shown that IL-2 has many functions of biological activity, inflammation and immune response, while IL-6 can activate leukocytes effectively and increase the expression of adhesion receptors in endothelial cells and astrocytes.^10^ TNF- alpha and hs-CRP can effectively stimulate the secretion of acute phase protein; increase the permeability of vascular endothelial cells, so that the expression of adhesion molecules and other inflammatory mediators can be induced. The above cytokines are inflammatory factors, and these cytokines can form enzymes through the release of astrocytes, increasing leukocyte infiltration and adhesion to vascular endothelial activity.^11^ In this study, the immune system stimulated by antigen of patients with brain injury can trigger immune system response, and the level of IL-2 in patients before treatment were significantly lower, while TNF-α, IL-6 and hs-CRP were significantly increased. Meanwhile, the inflammatory factors were also gradually changed with the decrease of the edema area around the blood clot after treatment. The postoperative IL-2 level of patients in research group were elevated and significantly higher than that in control group, while the IL-6, TNF-α and hs-CRP were decreased and significantly lower than that in control group. These results demonstrated that the neuroendoscopic minimally invasive surgery is less invasive than craniotomy, and it is conducive to the prognostic rehabilitation of patients.

SF is a labelled protein that appears to be excessively high as a marker of glial damage following hypertensive intracerebral hemorrhage and results in increased serum levels after it is released into the bloodstream. Hence, it can be used as a marker of impaired glial due to hypertensive intracerebral hemorrhage. SP showed a decreasing trend due to cerebral anoxia and cerebral edema caused by cerebral hemorrhage, meanwhile, with the decline of SP, other neurotransmitter metabolic disorder will also appear, thus the decrease of SP can aggravate the situation of edema and increase intracranial pressure.^12^ In this study, the SF levels of patients in the research group were significantly lower than that in the control group of patients with craniotomy. Moreover, the level of SP in the research group was obviously superior to that of the control group in craniotomy. The results exhibited that neuroendoscopic minimally invasive surgery can effectively promoted the recovery of damaged glial cells.

## CONCLUSION

It can be concluded that compared with craniotomy, minimally invasive surgery is more effective in treating hypertensive intracerebral hemorrhage, which is beneficial to restore neurological function, improve prognosis and reduce serum inflammatory factor level and has fewer complications.

### Authors Contribution

All authors contributed equally (Study design, Data collection, Manuscript preparation, Editing and Final approval).

### Abbreviations

**SP:** Serum substance P. **SSS:** Navier/Saint Stroke Scale.

**BI:** Barthel Index. **MRS:** Modified Rankin Scale.

**IL-6:** Interleukin-6. **hs-CRP:** High sensitive C-reactive protein.

**TNF-α:** tumor necrosis factor-α. **SF:** Serum Ferritin.

**CT:** Computed Tomography. **ECG:** Electrocardiography.
